# Human Bertielliasis in Amazonia: Case Report and Challenging Diagnosis

**DOI:** 10.1371/journal.pntd.0001580

**Published:** 2012-06-26

**Authors:** Adriano P. Furtado, Evander de J. O. Batista, Evonnildo C. Gonçalves, Anderson M. H. O. Silva, Francisco T. V. Melo, Elane G. Giese, Jeannie N. Santos

**Affiliations:** 1 Instituto de Ciências Biológicas, Universidade Federal do Pará, Pará, Brazil; 2 Núcleo de Medicina Tropical, Universidade Federal do Pará, Pará, Brazil; 3 Instituto de Saúde e Produção Animal, Universidade Federal Rural da Amazônia, Pará, Brazil; Emory University, United States of America

## Presentation of Case

In May, 2009, a 4-year-old girl from the town of Oriximiná, Pará state, Brazil, experienced 15 days of nocturnal abdominal pain, weight loss, abdominal distension, and the presence of “white worms” in the stool, but no diarrhea. At the local hospital, even though a conclusive diagnosis was not available, albendazole was prescribed for 5 days. Following this treatment, worms were still found in the patient's faeces, so the child was taken to the Tropical Medicine Nucleus of the Federal University of Pará (UFPA), where cestode infection was confirmed. This diagnosis was followed by a single oral dose of praziquantel (10 mg/kg).

During treatment, a further seven small fragments and one large portion of the worm's strobilus were expelled by the patient. The largest fragments were fixed in 10% formalin and others stored in saline solution, which were sent to the Cell Biology and Helmintology Laboratory at the UFPA for taxonomic analysis.

**Figure 1:**
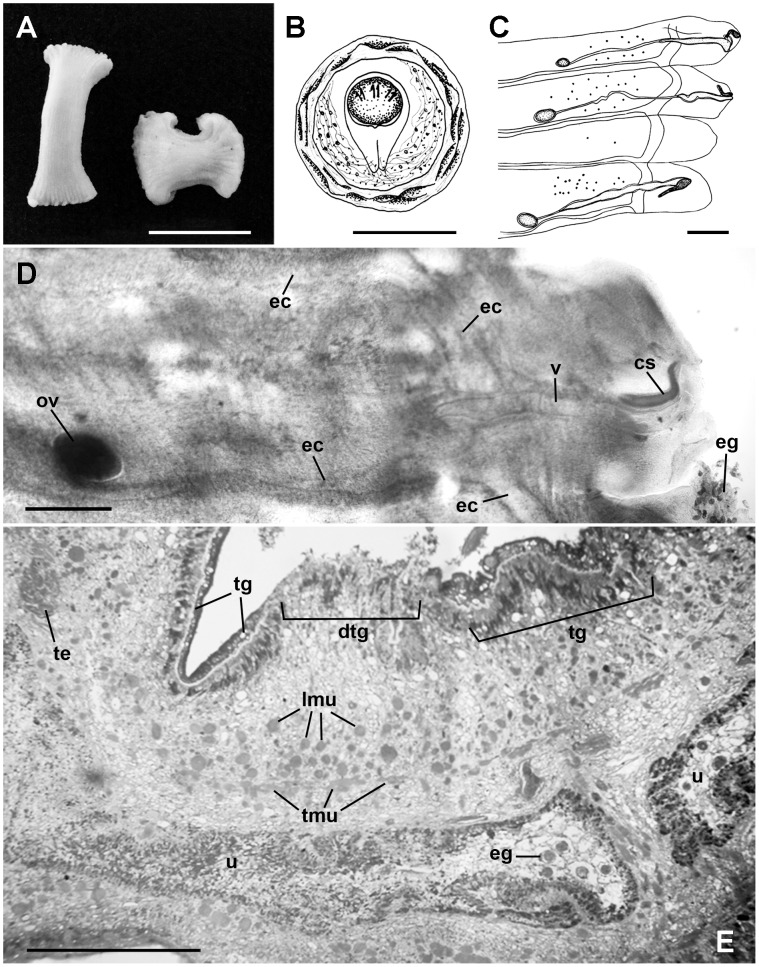
Morphological features of *Bertiella* sp. expelled by a 4-year-old girl of the Amazon region. (A) Macroscopic view of proglottids (Bar = 1 cm). (B) Egg illustration showing thick shell, albuminous layer, piriform apparatus, and oncosphere (Bar = 2.5 µm). (C) Mature proglottids illustration showing genital pores in an irregular, alternating arrangement, unlobed ovaries, vagina, cirrus sac, and excretory canals (Bar = 1000 µm). (D) Gravid proglottids under compression showing the ovary (ov), vagina (v), cirrus sac (cs), and excretory channels (ec). Numerous eggs (eg) were observed outside of proglottids (Bar = 500 µm). (E) Proglottids histological section, showing few eggs (eg) in the uterus (u). The tegument presented intact areas (tg) and discontinuous portions (dtg). Transversal (tmu) and longitudinal (lmu) muscles and testes (te) were also observed (Bar = 200 µm).

The smaller fragments were still moving when they arrived at the laboratory (Figure 1A), and were exuding a white substance, which was analyzed under a miscroscope. A large number of spherical eggs—0.042–0.047 µm (0.045 µm; *n* = 10)×0.041–0.046 µm (0.043 µm; *n* = 10)—were observed. Shell thickness 0.004–0.005 µm (0.005 µm; *n* = 10), albuminous layer 0.034–0.043 µm (0.037 µm; *n* = 10)×0.034–0.037 µm (0.035 µm; *n* = 10), piriform apparatus 0.024–0.31 µm (0.028 µm; *n* = 10)×0.015–0.018 µm (0.017 µm; *n* = 10), which is bifurcated at the tip, with contiguous filaments, and oncospheres 0.013–0.014 µm (0.014 µm; *n* = 10) with six hooks, characteristics of the Anaplocephalidae family (Figure 1B).

Five fragments were fixed under compression between two glass plates with AFA (2% acetic acid, 2% formaldehyde, and 93% 70% ethanol) for 24 hours. Some of the fragments were stained with carmine, and then dehydrated with ethanol, diaphanized in methyl salicylate, and mounted in Entellan. Under the microscope, 17–20 pregnant proglottids were observed per segment. The sides of the proglottids were not well defined, and appeared to be deteriorated.

The female reproductive system presented unlobed ovaries, 0.42–0.82 µm (0.58 µm; *n* = 10)×0.21–0.40 µm (0.30 µm; *n* = 10), located in the poral side of the proglottid; vagina 1.10–3.53 µm (2.30 µm; *n* = 9)×0.14–0.26 µm (0.16 µm; *n* = 9), opening into a common atrium with the cirrus sac, and extending at the excretory canals. Genital pores were in an irregular, alternating arrangement. Only the cirrus sac—0.53–0.74 µm (0.61 µm; *n* = 7)×0.09–0.16 µm (0.11 µm; *n* = 7)—of the male reproductive system was observed, and it was not possible to define the number of testicles, which further hampered the identification of the genus (Figure 1C and 1D).

The largest fragment of the strobilus was processed for setting in HistoResin. Sections of 3 µm were obtained and stained with 1% toluidine blue for histological analysis. Proglottids with a few eggs were observed in the uterus. Parasite tegument was damaged, presenting discontinuous portions. Few testes could be observed (Figure 1E).

**Figure 2:**
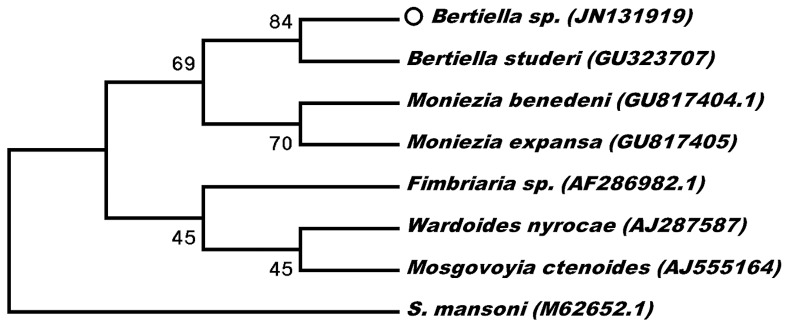
Phylogenetic tree between *Bertiella* sp. from this study (circle) and other Anoplocephalidae. Numbers in parentheses are GenBank accession numbers. *Schistosoma mansoni* were used as outgroup.

One of the fragments of the strobilus stored in saline solution was used for the molecular analysis of the 18S fragment of the ribosomal DNA, based on the protocol previously described [1]. The sequences obtained were edited manually and mixed using BioEdit [2] (GenBank accession number JN131919), and aligned with other 18S sequences of anoplocephalids available in GenBank using Mega 5 [3]. The phylogenetic analysis was based on Kimura's 2-parameter model for the neighbor-joining method, with 1,000 bootstrap replicates, which revealed that the sample collected in the present study was 84% similar to the sequence of *Bertiella studeri* (Figure 2).

### Consent for Publication

The patient's mother gave consent to have her case details published.

## Case Discussion

The Anoplocephalidae is a family of cestodes characterized by small suckers, proglottids wider than they are long, spherical eggs with piriform apparatus, and cysticercoid larvae that develop in arthropods. The genus *Bertiella* (Stilles & Hassall, 1902) includes a large number of species that parasitize mammals, including humans, in Africa, Asia, Australia, and the Americas [4]. Bertielliasis occurs when an intermediate host (Oribatidae mites) containing cysticercoid *Bertiella* larvae is ingested by the definitive host. The zoonotic infection of humans occurs in a similar way, principally in areas in which the population co-exists with non-human primates [4], [5].

The identification of *Bertiella* species is controversial, and since 1927, some authors have considered the geographic location of the host as a diagnostic criterion for classification [6]. Primates are considered to be infected by two *Bertiella* species—the type species, *Bertiella studeri* (Blanchard, 1891), which is common in the Eastern Hemisphere, and *Bertiella mucronata* (Meyer, 1895), which is found in the Western Hemisphere. Other species, such as *Bertiella satyri* (Blanchard, 1891) have been synonymized with *B. studeri* [7], although recently *B. satyri* was re-described as a valid species [8]. Reports of the infection of humans by *B. studeri* in the Western Hemisphere have been related to the transportation of infected monkeys between continents [6], [9].

The inadequate preparation and storage of samples of *Bertiella* obtained from humans normally leads to specimens that are inappropriate for morphological analysis, which results in frequent errors in the identification of taxa, and some authors have even suggested that *B. studeri* may be a complex of species [10].

Few cases of bertielliasis have been recorded in humans in the world. In South America, only seven cases of human bertielliasis have been reported, and all have been attributed to *B. mucronata* [5]. Three of these cases were recorded in Brazil, although the most recent report identified the parasite at only the genus level [11]. Until now, no human bertielliasis has been reported in the Amazon region and the risk of infection is not widely known, although *B. mucronata* has recently been observed in Peruvian titi monkeys [12].

Many practitioners are unaware of the poor performance of albendazole in intestinal cestodes. The identification of the genus through traditional taxonomic criteria was hampered by the prior application of praziquantel, which resulted in the fragmentation of the parasite's integument, and probable disruption of the internal organs. Despite these anatomical alterations, the morphometric analysis of the remaining intact structures indicated that they were consistent with those observed in the genus *Bertiella* [7].

The identification of the genus was confirmed based on the comparison of the DNA sequence obtained in the present study with those of other anoplocephalids, although it was not possible to compare with other sequences of *Bertiella* genus (the single 18S sequence of anoplocephalids available was from *B. studeri*). This emphasizes the importance of molecular biology as a complementary diagnostic tool where morphological analyses are inconclusive. To our knowledge, the nucleotide sequence presented here is the first described from the Americas, and may not only provide an important baseline for the analysis of future cases in the region, but may also be useful for phylogenetic studies.

This is the fourth report of bertielliasis in humans from Brazil, and the first from the Amazon region. A factor that may have contributed to the infection of the patient is that the girl and her family were regular visitors to a farm inhabited by capuchin monkeys (*Cebus* sp.).

The encroachment of forests by human populations may expose them to complex ecological relationships. This is especially clear in the Amazon region, where populations, because of custom or necessity, are intimately related with the forest, even though this may expose them to a series of poorly known, emerging parasitic diseases, as shown in a number of cases recorded recently [13], [14].

**Key Learning Points**

- *Bertiella* is a genus of the Anoplocephalidae family that includes parasites of mammals that can infect humans.
- The specific identification is based on morphological features and geographical localization of infection.
- Molecular approaches with phylogenetic relationships of genetic sequences are tools that can be used in similar cases.
